# Taxol-Loaded MSC-Derived Exosomes Provide a Therapeutic Vehicle to Target Metastatic Breast Cancer and Other Carcinoma Cells

**DOI:** 10.3390/cancers11060798

**Published:** 2019-06-09

**Authors:** Catharina Melzer, Vanessa Rehn, Yuanyuan Yang, Heike Bähre, Juliane von der Ohe, Ralf Hass

**Affiliations:** 1 Biochemistry and Tumor Biology Lab, Department of Obstetrics and Gynecology, Hannover Medical School, 30625 Hannover, Germany; melzer.catharina@mh-hannover.de (C.M.); Vanessa@Rehn-wennigsen.de (V.R.); kateyang-y@hotmail.de (Y.Y.); Ohe.Juliane.von.der@mh-hannover.de (J.v.d.O.); 2 Tongji Hospital Affiliated Tongji University, Shanghai 200065, China; 3 Institute of Pharmacology, Hannover Medical School, 30625 Hannover, Germany; Baehre.Heike@mh-hannover.de; 4 Research Core Unit Metabolomics, Hannover Medical School, 30625 Hannover, Germany

**Keywords:** therapeutic exosomes, mesenchymal stem cells, targeted therapy, cancer cells, tumor therapy

## Abstract

MSC-derived exosomes display, among others, an efficient biocompatibility and a reduced intrinsic immunogenicity, representing a valuable vehicle for drug delivery in a tumor-therapeutic approach. Following treatment of several human mesenchymal stroma/stem-like cell (MSC) populations with sub-lethal concentrations of taxol for 24 h, exosomes were isolated and applied to different human cancer populations including A549 lung cancer, SK-OV-3 ovarian cancer, and MDA-hyb1 breast cancer cells. While MSC control exosomes revealed little if any growth inhibition on the tumor cells, exposure to taxol-loaded MSC-derived exosomes was associated with 80–90% cytotoxicity. A similar application of taxol-loaded exosomes from HuVEC displayed much fewer effects. Quantification by LC-MS/MS analysis demonstrated a 7.6-fold reduced taxol concentration in MSC exosomes when compared to equivalent cytotoxic in vitro effects achieved with taxol substances, indicating a specific and more efficient tumor-targeting property. Consequently, MSC-derived taxol exosomes were tested in vivo. Highly metastatic MDA-hyb1 breast tumors were induced in NODscid mice, and systemic intravenous application of MSC-derived taxol exosomes revealed a more than 60% reduction of subcutaneous primary tumors. Moreover, the amount of distant organ metastases observed at least in lung, liver, spleen, and kidney was reduced by 50% with MSC taxol exosomes, similar to the effects observed with taxol, although the concentration of taxol in exosomes was about 1000-fold reduced. Together, these findings in different cancer cell populations and in vivo provide promising future perspectives for drug-loaded MSC-derived exosomes in efficiently targeting primary tumors and metastases by reducing side effects.

## 1. Introduction

Extracellular vesicles (EVs) represent membranous organelles generated by various cells under different physiological and pathophysiological conditions and can be discriminated into, among others, exosomes, microvesicles, apoptotic/necroptotic bodies, phagosomes, and damage-associated molecular patterns (DAMPs) [1,2,3]. Besides very small exomers, EVs differ in size, origin, and content and may be separated together with lipid-based but non-vesicular structures such as chylomicrons or very-low-, low-, intermediate-, and high-density lipoproteins [4].

Exosomes as small membrane particles approximately 20–200 nm in diameter represent multivesicular bodies of endocytic origin released into the extracellular compartment and contain a large panel of proteins, mRNAs, and regulatory microRNAs (miRs), which can alter the functionality of recipient cells [5,6]. Consequently, the EV secretome changes significantly in disease, inflammation, and cancer [7]. Typical marker proteins of exosomes include at least surface glycoproteins of the tetraspanin transmembrane-4 family such as CD9, CD63, and CD81 (=TAPA-1 (target of the antiproliferative antibody 1) = tetraspanin-28) [5,8].

Most cells release exosomes, including cancer cells and populations that can associate with tumor tissue, such as heterogeneous mesenchymal stroma/stem-like cells (MSC), also termed multipotent mesenchymal stromal cells or medicinal signaling cells [9,10]. According to multiple direct and indirect interactions between MSC and cancer cells, mutual exchange of exosomes contributes to altering cancer cell functionalities and vice versa to modify MSC into carcinoma-associated (CA-) MSC [11,12,13,14].

MSC preferentially reside in perivascular niches of nearly all kinds of human tissues [15,16] and exhibit certain functional differences according to their tissue-specific origins, although heterogenic MSC populations share distinct surface marker expressions such as CD73, CD90, and CD105 by maintenance of the capability to differentiate at least along certain phenotypes of the mesodermal lineage [17,18,19,20]. Moreover, MSC contribute to the regulation of hematopoietic stem cell homeostasis in the bone marrow and accumulate at damaged or injured tissues to utilize repair processes [21] and support neovascularization [22]. MSC are also located in a tumorigenic microenvironment and contribute to immune modulation [23], tumor angiogenesis, and alteration of a large variety of cancer cell functionalities [14,24,25,26,27] by different types of interactions including the release of therapeutically useful exosomes [28].

Both MSC and cancer cells including cancer stem-like cells can secrete extracellular vesicles with mutual metabolic effects on tumorigenesis, whereby exosomes from heterogeneous MSC of different tissue origin contain various unique factors displaying distinct functionalities in tumors [29,30,31]. Thus, MSC-derived exosomes can mediate distinct effects in breast cancer cells including suppression of angiogenic potential by down-modulation of VEGF via exosome-associated miR-16 [32]. Moreover, miR-222/223-containing exosomes released by tumor-stimulated MSC confer drug resistance and promote quiescence in breast cancer cells [33]. Conversely, human umbilical cord MSC-derived exosomes can protect against cisplatin-induced nephrotoxicity and promote cell proliferation [34]. Other research has demonstrated that human bone marrow MSC-derived exosomes increase tumor growth in vivo [35]. Irrespective of these controversial findings transcellular exchange of exosomes provides a useful tool to deliver therapeutic compounds since the biocompatibility of exosomes enables drug transport as a cell-free vehicle that is capable of overcoming various biological barriers and may target various cancer cell populations including cancer hybrid cells and cancer stem-like cells [13,36,37,38]. Accordingly, the present study provides data for such an innovative therapeutic approach after the isolation of EVs with exosomal properties from taxol-exposed MSC followed by subsequent treatment of various in vitro cancer populations and in vivo tumors with metastases.

## 2. Results

**Figure 1:**
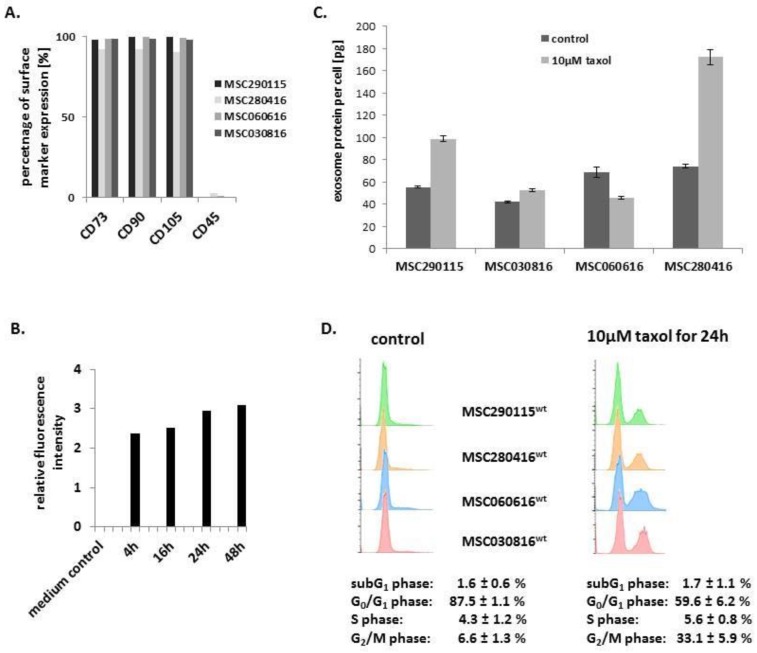
(**A**) Characterization of four different mesenchymal stroma/stem-like cells (MSC) populations (MSC280416 P2; MSC290115 P3; MSC030816 P4; MSC060616 P6) was performed by flow cytometry analysis with the positive markers CD73, CD90, and CD105 as compared to the negative marker CD45. (**B**) Production of exosomes and release into serumfree medium by MSC^GFP^ was analyzed at the time points indicated. Relative fluorescence intensity was measured in exosome aliquots whereby auto-fluorescence of cell-free supernatant (medium control) was subtracted (normalized to medium control = 0). (**C**) Protein quantification was performed in cell lysates of the four MSC primary populations. Moreover, protein was also quantified in exosomes from these MSC, respectively, isolated from 24 h serumfree supernatant of steady state cultures (control) and from 24 h serumfree supernatant of previously 10 µM taxol-treated cells for another 24 h. The amount of exosomal protein per cell is calculated in the bar diagram. Data represent the mean ± s.d. of three replicates. (**D**) Cell cycle analysis of the four different control MSC (left histograms) and after treatment with 10 µM taxol for 24 h (right histograms). Quantification of cell cycle phases was performed using FlowJo V10 cell cycle software.

Previous work has demonstrated uptake and incorporation of MSC-derived exosomes by cancer cells [28]. Accordingly, we addressed the question whether these MSC-derived exosomes could be used as a vehicle to deliver chemotherapeutic compounds to cancer cells. To minimize donor-specific effects, four different human MSC populations in distinct passages (MSC280416 P2; MSC290115 P3; MSC030816 P4; MSC060616 P6) were randomly chosen for exosome production. Evaluation of these primary four MSC cultures revealed adherence and constitutive expression of the core markers CD73, CD90, and CD105 in more than 90% of the individual cell populations, respectively, with simultaneous low to undetectable levels of CD45 consistent with MSC characterization guidelines [15,18,20] (Figure 1A).

Supernatants of 24 h serumfree MSC cultures were sequentially centrifuged according to the described exosome isolation method. Analysis by transmission electron microscopy as described elsewhere [39] revealed exosome-like round organelles that varied in size between 50 and 200 nm (Supplementary Material Figure S1). The kinetic of exosome production by MSC^GFP^ was investigated after preparation of serumfree medium at different time points and demonstrated a progressively increasing exosome release, which reached a plateau after 24 h with little if any change after 48 h (Figure 1B). Accordingly, collection of MSC-derived exosomes was systematically performed from 24 h serumfree stem cell cultures. Quantification of exosomes by BCA protein assay demonstrated slight differences among the various MSC types, and equal aliquots of the randomly chosen four populations revealed an average of 113.0 ± 29.9 µg exosome protein produced by 1.89 ± 0.21 × 10^6^ MSC, which equals 60 ± 14.3 pg exosome protein per control cell within 24 h (Figure 1C). Likewise, taxol-treated MSC exhibited an average of 134.8 ± 67.1 µg exosome protein produced by 1.68 ± 0.7 × 10^6^ MSC, which equals 92.4 ± 58.2 pg exosome protein per taxol-treated cell within 24 h (Figure 1C).

Exosomes from the four selected MSC cultures were isolated from control MSC in steady state and from MSC after treatment with 10 µM taxol as the maximal tolerable concentration for 24 h. This was substantiated by cell cycle analysis of all four MSC populations, demonstrating 87.5% ± 1.1% of control cells in G0/G1 and 6.6% ± 1.3% in G2/M phase after 24 h in serumfree medium (Figure 1D, left histogram). Conversely, taxol treatment and subsequent 24 h culture in serumfree medium was associated with a reduced G0/G1 phase of 59.6% ± 6.2% and an approximately 5-fold accumulation of 33.1% ± 5.9% in G2/M phase (Figure 1D, right histogram). However, apoptotic/necroptotic subG1 phase cells remained at equally low levels in control (1.6% ± 0.6%) and taxol-treated (1.7% ± 1.1%) MSC populations, confirming no detectable cytotoxic effects (Figure 1D).

**Figure 2:**
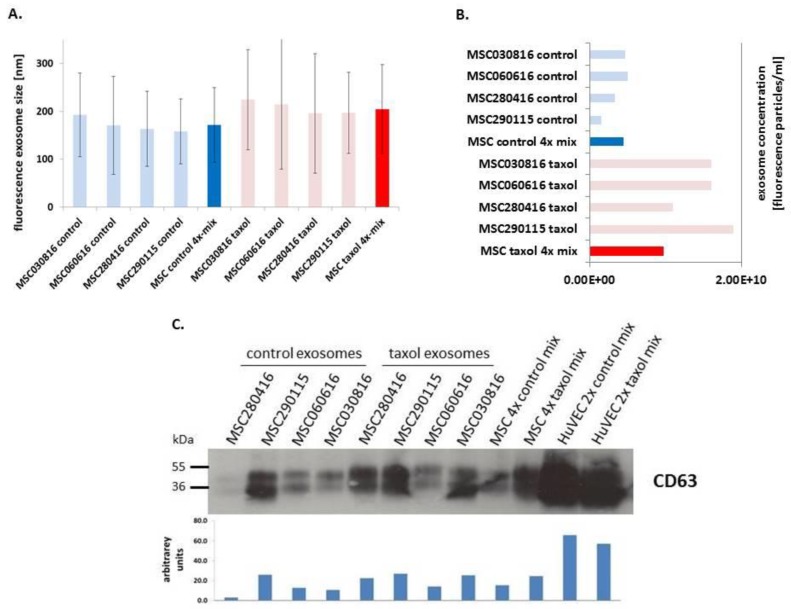
Characterization of isolated exosomes by nanoparticle tracking analysis (NTA) was performed in fluorescence mode of the ZetaView PMX120 by measurement of (**A**) fluorescence particle size and (**B**) the amount of fluorescence particles from the four different MSC cultures (light blue bars), a mixture of the four MSC exosomes (blue bars), the four different MSC cultures after treatment with 10 µM taxol for 24 h (light red bars), and a mixture of the four MSC-derived fluorescence particles after treatment with 10 µM taxol for 24 h (red bars), respectively. (**C**) Immunoblot analysis (upper panel) was performed for the presence of tetraspanins (CD63) in the various different MSC exosome preparations in the absence and presence of taxol as indicated, a mixture of two different HuVEC control exosome preparations, and a particle mixture of these two HuVEC cultures after treatment with 10 µM taxol for 24 h. Intensities of the appropriate expression levels (lower panel) were quantified using the ImageJ software. The corresponding original blot is documented in Supplementary Materials Figure S5.

Characterization and quantification of isolated vesicles for exosomal properties was performed by nanoparticle tracking analysis (NTA) and by Immunoblot analysis (Figure 2). Exosome preparations usually display a negative surface charge as determined by the zeta potential, whereby higher charged particles are less likely to aggregate and are much more stable in dispersion [3]. Accordingly, a reasonable quality and stability of our exosomes preparations was confirmed by measuring the surface charge zeta potential and particle mobility at 25 °C, respectively. An equal mixture of the four MSC control exosomes exhibited a zeta potential of −40.19 ± 1.67 mV compared to −43.08 ± 1.58 mV of the four MSC taxol-treated exosomes. In addition, the four MSC control exosomes demonstrated a particle mobility of −3.39 ± 0.14 µm/sec/V/cm compared to −3.63 ± 0.13 µm/sec/V/cm in MSC taxol-treated exosomes.

Whereas all measurements slightly increased between the NTA light scatter and the NTA fluorescence values, the latter were considered as exosome diameters due to the GFP-labeling of the cells. Thus, an equal mixture of GFP-labeled exosomes derived from the four MSC populations revealed an average size of 171.4 ± 78.2 nm. Moreover, exosomes isolated from the 24 h taxol-treated MSC exhibited an average diameter of 204 ± 93.1 nm, suggesting that incubation with taxol slightly enlarged the corresponding exosomes, although not statistically significantly (Figure 2A). Exosome concentrations revealed 4.5 × 10^9^ fluorescence particles/mL in control MSC and 9.8 × 10^9^ fluorescence particles/mL in taxol-treated MSC, indicating a significantly increasing exosome release per cell (*p* < 0.001) upon exposure to this chemotherapeutic compound, which is most likely related to enhanced cellular stress upon taxol treatment (Figure 2B). Indeed, cellular stress including heat was associated with elevated production of doxorubicin-loaded exosomes [40]. Further exosome analysis by the presence of tetraspanins in immunoblots revealed altered expression levels of the 26 kDa core protein and the 30–60 kDa glycosylated form of CD63 in all control and taxol-treated samples, with ImageJ quantification for relative intensities (Figure 2C). This is supported by Western blot analysis of previous work demonstrating the presence of exosome-associated CD63 tetraspanin molecules in MSC-derived exosome preparations [28].

Together, these data substantiated isolation of EVs displaying stable exosomal properties.

**Figure 3:**
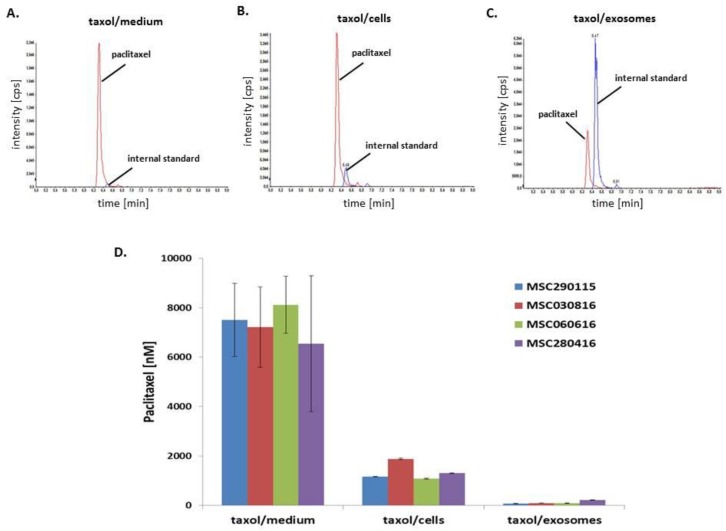
Representative MSC290115^GFP^ LC-MS/MS-chromatograms of paclitaxel (m/z: 854→105, retention time: 6.3 min) and the internal standard docetaxel (m/z: 808→226, retention time: 6.5 min) of (**A**) cell culture medium supernatant of 10 µM taxol-treated MSC290115 after 24 h, (**B**) cell lysate of 10 µM taxol-treated MSC290115 after 24 h, and (**C**) exosome lysate released after 24 h from previously 10 µM taxol-treated MSC290115 for 24 h. The peak of the internal standard represents constant intensities in the range of 5 × 10^4^ cps in all samples. Accordingly, paclitaxel intensities are varying in the different samples whereby in (**A**). 2.2 × 10^6^ cps, in (**B**). 3.5 × 10^5^ cps, and in (**C**). 2.5 × 10^4^ cps were determined. (**D**) Quantification of taxol concentrations were performed by LC-MS/MS in the corresponding cell culture medium supernatants of 10 µM taxol-treated four MSC populations (MSC290115^GFP^, MSC030816^GFP^, MSC060616^GFP^, and MSC280416^GFP^) (=taxol/medium), in the corresponding cell lysates of 10 µM taxol-treated four MSC populations (=taxol/cells), and in the corresponding exosome lysates released after 24 h from previously 10 µM taxol-treated four MSC populations for 24 h (=taxol/exosomes). Data represent the mean ± s.d. of three replicates.

Determination and quantification of the amount of taxol in the MSC-derived exosomes delivered to the cancer cells was assessed by LC-MS/MS. Representative histograms for the detection of taxol (paclitaxel) and its evaluation compared to the internal standard (docetaxel) are presented for 1.68 × 10^5^ MSC290115^GFP^ and demonstrated 7.5 ± 1.5 µM taxol (*n* = 3) in the cell culture medium supernatant remaining after a 24 h treatment with 10 µM taxol (Figure 3A,D). Moreover, cell-associated taxol of MSC290115^GFP^ exhibited 1.17 ± 0.01 µM (*n* = 3) after a 24 h stimulation with 10 µM taxol (Figure 3B,D) and released exosomes isolated after further 24 h culture in serumfree medium of previously 24 h-treated MSC290115^GFP^ with 10 µM taxol revealed 74.9 ± 3.9 nM (*n* = 3) of this compound (Figure 3C,D).

The minimalized heterogeneity of taxol incorporation into MSC by randomly choosing four different donors and passages is summarized in Figure 3D. Thus, 7.3 ± 0.7 µM taxol (*n* = 4) remained in the medium supernatant of the four MSC cultures after a 24 h treatment with 10 µM taxol. Accordingly, 1.4 ± 0.4 µM taxol (*n* = 4) was found in the cell homogenates of the four taxol-treated MSC cultures demonstrating 14% incorporation of taxol. Furthermore, 123 ± 0.7 nM taxol (*n* = 4) was detectable in the different exosome preparations of the four taxol-exposed MSC populations equivalent to 1.23% incorporation of the initial taxol stimulation (Figure 3D).

**Figure 4:**
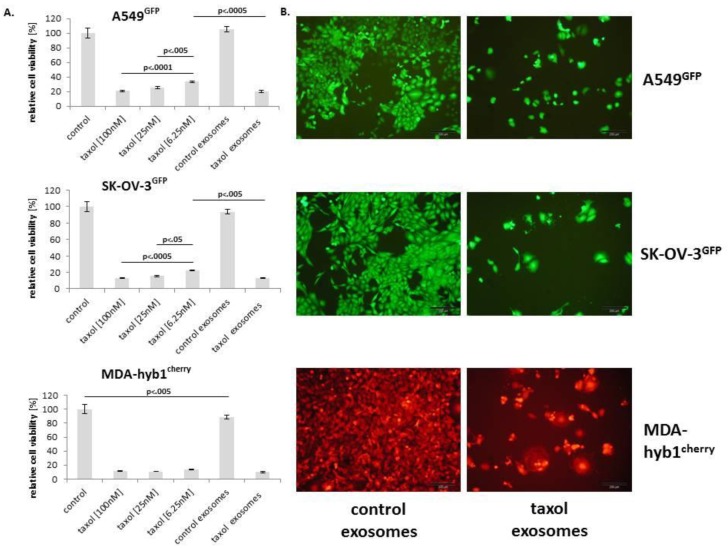
(**A**). Relative chemotherapeutic response of different human cancer cell populations, including A549^GFP^ lung cancer (upper panel), SK-OV-3^GFP^ ovarian cancer (middle panel), and MDA-hyb1^cherry^ breast cancer (lower panel) cells was tested for relative cell viability after exposure to different concentrations of taxol (100 nM, 25 nM, and 6.25 nM) compared to the appropriate steady state cancer cell populations cultured in the highest solvent concentration of taxol (control). Moreover, relative cytotoxic effects of control exosomes (from steady state MSC cultured in the highest taxol solvent concentration) and of taxol exosomes (1:150 dilution derived from 10 µM taxol-treated MSC) were evaluated. Following 72 h incubation of the different cancer cell population data were obtained from fluoroscan assays in triplicate and calculated as mean ± s.d. with corresponding controls set to 100%. Significance (*p*) was calculated by ANOVA followed by Tukey´s multiple comparisons test. (**B**) Fluorescence microscopy was performed for the different cancer cell lines following incubation with control exosomes (left micrographs) and compared to treatment with taxol exosomes (right micrographs) for 72 h. Bars represent 200 µm.

Equal aliquots from all four MSC-derived control or taxol exosomes were combined and incubated with different cancer cell populations, including A549 lung cancer cells, SK-OV-3 ovarian cancer cells, and MDA-hyb1 breast cancer cells. Cytotoxic effects were evaluated by fluorescence reduction in a fluoroscan assay whereby culture of the different cancer cell populations in control medium was set to 100%. Treatment with different amounts of taxol exhibited a concentration-dependent cytotoxicity of all cancer cells. Thus, exposure to 100 nM taxol for 72 h revealed a viability of 21.2 ± 1.2% (*n* = 3) in A549 lung cancer cells, 12.7 ± 0.3% (*n* = 3) in SK-OV-3 ovarian cancer cells, and 11.3 ± 0.8% (*n* = 3) in the MDA-hyb1 hybrid breast cancer variant (Figure 4A). Conversely, incubation with MSC-derived control exosomes (1:150 dilution) demonstrated fluorescence levels similar to A549 lung cancer cells and SK-OV-3 ovarian cancer control cells with little effects on MDA-hyb1 cells reaching about 90% of the growth and viability rate of untreated control cells. In contrast, treatment with exosomes derived from taxol-treated MSC (1:150 dilution) carrying 0.82 nM taxol (=1.23 nM/150) was associated with a pronounced cytotoxicity in all different cancer types which was significantly elevated as compared to effects observed with the 6.25 nM taxol substance (Figure 4A). These findings suggested that MSC-derived taxol exosomes displayed a markedly enhanced tumor cell killing efficiency, albeit carrying 7.6-fold less taxol, which was also supported by morphological alterations in fluorescence microscopy. Whereas exposure to control exosomes revealed a normal viable phenotype in the different cancer cell populations including A549^GFP^ lung cancer, SK-OV-3^GFP^ ovarian cancer, and MDA-hyb1^cherry^ breast cancer cells (Figure 4B, left panel), a significantly reduced cell number accompanied by apoptotic/necroptotic disintegration of the different cancer cell populations was observed within 72 h following treatment with MSC taxol-primed exosomes (Figure 4B, right panel). These findings suggested a successful in vitro treatment of different cancer cell populations with taxol-treated MSC-derived exosomes.

To address the question of whether MSC-derived exosomes exhibit a potentially superior advantage for tumor targeting, two different HuVEC populations were randomly chosen as a comparison and similarly tested. Primary HuVECs as endothelial cell model were selected due to the supportive role of endothelial cells within the tumor microenvironment to initiate angiogenesis and promote subsequent tumor neovascularization. Characterization of these primary HuVEC cells was performed by FACS analysis revealing more than 99% expression of the endothelial marker CD31 paralleled by undetectable levels of CD90 (Supplementary Material Figure S2). Treatment of the two HuVEC cultures with 10 µM taxol for 24 h revealed little if any differences in the appearance of dead cells similar to the observations in the different MSC populations. Characterization of the exosome preparations from the two control HuVEC cultures by NTA demonstrated an average diameter of 152.1 ± 65.2 nm for HuVEC-4 and 159.8 ± 74.5 nm for HuVEC-6. In agreement with the observations in the four different MSC populations, the exosome size in HuVECs slightly increased after taxol treatment and accordingly, 10 µM taxol treatment for 24 h revealed an average diameter of 160.6 ±7.4 nm in HuVEC-4 and 169.7 ± 74.3 nm in HuVEC-6 (Supplementary Material Figure S3). Compared to MSC, however, the amount of released exosomal particles in HuVECs was increased by about 36-fold reaching 1.65 × 10^11^ particles/mL. While taxol exposure of HuVECs reduced the release of exosomes to 1.02 × 10^11^ particles/mL, this amount of exosomes from taxol-treated HuVECs was still 10.4-fold enhanced in comparison to the particles detected from taxol-treated MSC (Supplementary Material Figure S4). Accordingly, HuVEC-derived exosomes demonstrated the expression of tetraspanins with significantly higher levels of CD63 as compared to MSC-derived exosomes (Figure 2C).

**Figure 5:**
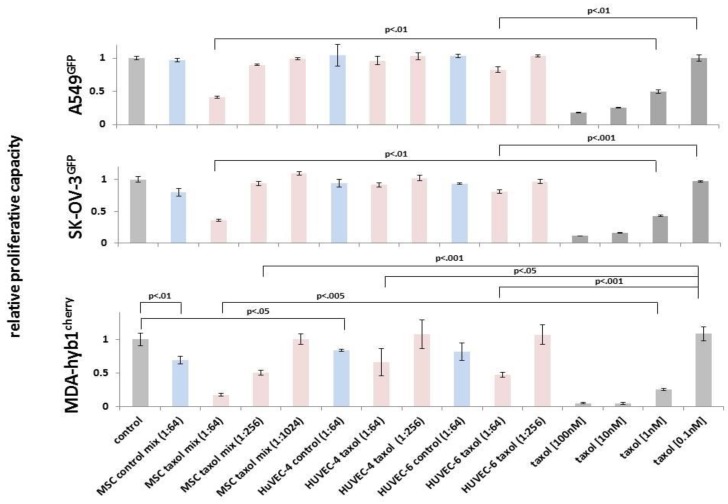
In vitro cytotoxicity of different human cancer cell lines including A549 lung cancer (upper panel), SK-OV-3 ovarian cancer (middle panel), and MDA-hyb1 breast cancer (lower panel) was measured by GFP fluoroscan assay. The different cancer cell populations were treated with appropriate dilutions of control exosomes from MSC and HuVECs as indicated (light blue bars), taxol-loaded exosomes from MSC and HuVECs as indicated (light red bars), and taxol substance (0.1 nM to 100 nM, dark grey bars) for 72 h, respectively. Fluorescence values of untreated steady state control cells (control, light gray bars) were set to 1, and relative cytotoxic effects were calculated as part of the control. Data represent the mean + s.d. (*n* ≥ 3), and significance (*p*) was calculated by an unpaired two-tailed Student’s *t*-test.

For evaluation of a potentially superior effectiveness, we applied HuVEC exosomes in comparison to MSC-derived exosomes to the different cancer cell populations in a concentration-dependent manner. While a 1:64 dilution of taxol exosomes from MSC reduced the amount of A549 lung cancer cells by 59%, corresponding taxol exosomes (1:64) from HuVEC-6 reached only a 17% reduction and little if any effects were observed with taxol exosomes (1:64) from HuVEC-4 after 72 h. Treatment of A549 with 1 nM and 10 nM taxol substance reached about 50% and 74% of A549 cytotoxicity, respectively (Figure 5, upper panel).

Similarly, exposure to MSC-derived taxol exosomes (1:64) revealed a 64% reduction of SK-OV-3 cells compared to only 18% with taxol exosomes (1:64) from HuVEC-6 and no significant effect with taxol exosomes (1:64) from HuVEC-4. Taxol substance (1 nM and 10 nM) revealed a 57% and 84% reduction of the ovarian cancer after 72 h, respectively (Figure 5, middle panel).

While control exosomes (1:64) slightly reduced the amount of MDA-hyb1 cells (from MSC by about 30% and from HuVEC-4 by about 17%), treatment of MDA-hyb1 cells with MSC-derived taxol exosomes (1:64) was associated with an 83% cytotoxicity, and a 1:256 dilution still reached a 50% reduction of these cancer cells after 72 h. HuVEC-derived taxol exosomes were much less effective, revealing only a 34% cytotoxicity with HuVEC-4 exosomes (1:64) and a 53% cytotoxicity with HuVEC-6 exosomes (1:64). In contrast, a 1:256 dilution of taxol exosomes from both HuVEC populations displayed no detectable effects. Treatment of these breast cancer cells with 1 nM and 10 nM taxol substance reached about 74% and 99% cytotoxicity, respectively (Figure 5, lower panel).

Although standardized exosome potency assays and definitions for a comparable bioequivalence remain to be established, together, these in vitro data suggested a promising approach for MSC-derived exosomes as a chemotherapeutic vehicle with superior effects as compared to an even 10.4-fold higher amount of HuVEC-derived exosomes. A possible explanation for the superiority of MSC exosomes may be attributed to the multipotent and unique functional properties of these stem-like cells, which are not observed in HuVECs or other cell populations.

**Figure 6:**
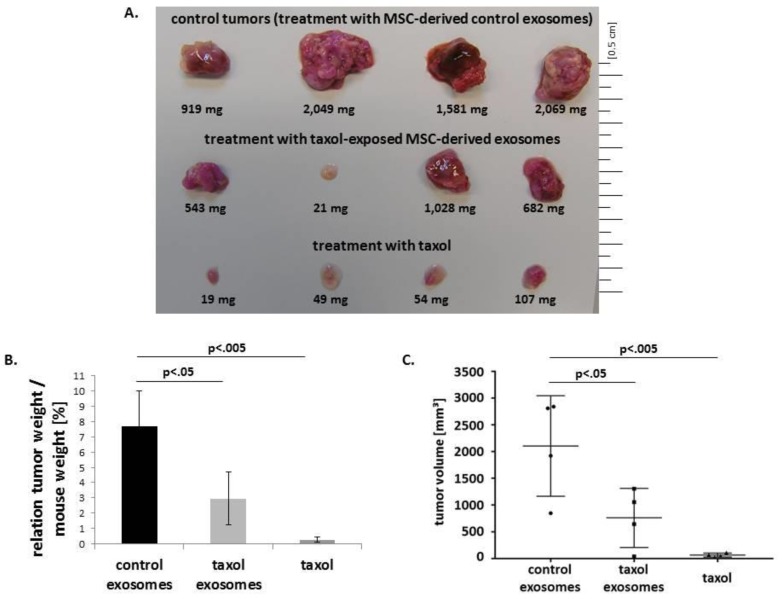
Tumors were grown in NODscid mice after injection of human mcherry-labeled MDA-hyb1 breast cancer cells. (**A**) Tumor weight of each treatment group (*n* = 4) was measured after dissection of the solid subcutaneous primary tumors only (excluding metastatic tumor tissue). (**B**) The mouse weight was determined and the ratio of tumor weight to the corresponding mouse weight was calculated. Data represent the mean ± s.d. and significance (*p*) was calculated by ANOVA followed by Dunnett´s multiple comparisons test. (**C**) Tumor length and width of each tumor was measured and the average tumor volume was calculated for each treatment group according to [41]. Data represent the mean ± s.d. and significance (*p*) was calculated by ANOVA followed by Dunnett´s multiple comparisons test.

We therefore tried to substantiate these findings with MSC-derived exosomes in vivo by using the aggressively metastasizing MDA-hyb1 breast cancer cells for induction of tumors in NODscid mice. Following detectable tumor development in 12 NODscid mice within 5 d, treatment was performed in three groups twice weekly by appropriate tail vein injection of 1) 100 µL of exosomes isolated from solvent (control), 2) 100 µL of taxol-treated MSC containing an average concentration of 123 nM taxol, or 3) 100 µL of 117 µM taxol substance. Each mouse was treated four times with taxol substance or MSC-derived exosomes (either from control MSC or taxol-treated MSC), respectively, following isolation from approximately 1.7 × 10^6^ MSC/application and the same MSC-derived exosome mixture as used in the in vitro cancer cell assay (Figure 4). After 21 d, animals were sacrificed by cervical dislocation and dissection of the tumors revealed an average tumor weight of 1,655 ± 467 mg (*n* = 4) in control animals (after intravenous injection of MSC-derived control exosomes) (Figure 6A). Following treatment with exosomes isolated from previously taxol-incubated MSC, the average tumor weight was reduced by 64.2%, reaching 593 ± 394 mg (*n* = 4). Substance control was achieved by intravenous application of initially 10 mg/kg taxol and thereafter, due to incompatibility, 5 mg/kg taxol twice weekly, which displayed a final tumor weight of 57 ± 32 mg (*n* = 4) equivalent to a 96.5% reduction compared to the control tumors (Figure 6A).

Similar data were obtained by comparing the relationship of tumor weight to mouse weight. This value decreased from 7.7 ± 2.3 in control tumors by 62.3% to 2.9 ± 1.7 in taxol exosome-treated tumors and further down to 0.3 ± 0.1 in taxol-treated tumors (Figure 6B). These findings were also substantiated by the evaluation of tumor volume. Tumor volumes of MDA-hyb1-induced tumors were calculated with the longitudinal diameter (length) and the transverse diameter (width) [41]. Accordingly, the average control tumor volume of 2103 ± 815 mm^3^ decreased by 63.9% to 759 ± 477 mm^3^ in taxol exosome-treated tumors and to 66 ± 32 mm^3^ in taxol-treated tumors (Figure 6C). These data documented that a four-time systemic application of exosomes from only about 2 × 10^6^ previously taxol-treated MSC significantly reduced mouse tumor growth by more than 60%. 

**Figure 7:**
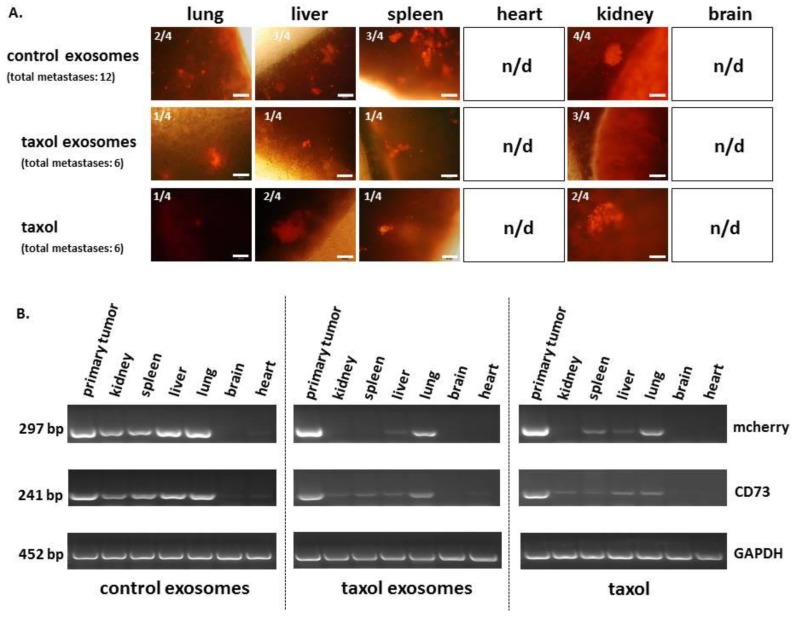
**(A**) Formation of distant organ metastases was detected by appearance of mcherry fluorescence in thin sections of organ tissues. Exemplary phase contrast/luorescence microscopy overlay pictures of organ metastases are documented. Moreover, the total number of organs with distal metastatic tumor cells is displayed (n.d. = not detectable). Bars represent 100 µm. (**B**) PCR analysis of primary tumor and organ tissue aliquots was performed to substantiate detection of metastatic MDA-hyb1 cancer cells by the presence of mcherry and CD73 transcripts. Unaltered GAPDH mRNAs served as loading control.

Detection and formation of distant organ metastases became evident by thin section cherry fluorescence microscopy (Figure 7A). Whereas no metastases were observed in heart or brain, respectively, therapeutic effects of taxol-loaded exosomes were clearly detectable during metastatic growth. Thus, a total of 12 organ metastases (lung, liver, spleen, and kidney) in control mice were reduced to six identified metastases in taxol exosome-treated mice. The same number of six total organ metastases was detectable in mice after application with maximal doses of taxol (Figure 7A). 

These observations were also supported by RT-PCR, demonstrating simultaneous expression of the mcherry gene originating from the MDA-hyb1 breast cancer cells in the representative organs. Moreover, treatment with taxol-loaded exosomes or taxol revealed a reduced mcherry expression, whereby unaltered GAPDH expression served as a loading control. In addition, previous microarray and PCR data demonstrated expression of CD73 in the MDA-hyb1 cells [25]. These findings were used to further substantiate the presence of CD73-carrying MDA-hyb1 breast cancer cells in the primary tumor and at reduced expression levels in organ metastases of kidney, spleen, liver, and lung, however, these cells were undetectable in brain and heart tissue (Figure 7B). In addition, quantification of taxol as pM per gram [g] wet tissue revealed about 43.6 pM/g tumor tissue in taxol-treated mice as compared to a 29-fold reduced amount of 1.5 pM/g in taxol exosome-treated tumors. Furthermore, taxol in metastatic tissues of kidney and liver was below the detection limit in taxol exosome-treated mice. Conversely, taxol-treated mice exhibited 0.4 pM/g taxol in the kidney and 4.3 pM/g taxol in the liver. 

Altogether, these findings demonstrate a significant reduction of both primary tumor growth and the appearance of organ metastases by an approximately 1000-fold reduced taxol concentration using a systemic approach with therapeutic MSC-derived exosomes. Moreover, these data suggested an about 34-fold more specific addressing, delivery, and accumulation of taxol to these tumors via taxol exosomes since the initially 1000-fold reduced amount of taxol administered by taxol exosomes to the whole mice was reduced by only 29-fold when quantified in the tumor tissues.

## 3. Discussion

MSC-mediated secretion of exosomes within the tumor microenvironment enables them to relay tumor-promoting and/or tumor-suppressive signals by carrying distinct surface molecules. Controversial findings discuss MSC and their secreted products with tumor inhibitory [42,43] or tumor-promoting properties [44,45,46]. While MSC subtypes exhibit an alternating balance between cancer-promoting (CA^+^-MSC) and cancer-inhibiting (CA^–^-MSC) functionalities, the overall effects were suggested to partially depend on the net balance between these opposite functioning cell-derived exosomes [13].

The usage of exosomes in clinical applications, particularly in anti-cancer therapy is still at the beginning [47]. While physiological functions of MSC-derived exosomes are not defined, the present study demonstrated partial cytotoxic effects of MSC- and HuVEC-released control exosomes after application to triple negative human MDA-MB-231-derived MDA-hyb1 breast cancer cells, suggesting various exosomal anti-tumor content including distinct proteins, DNA, mRNAs and miRs [48]. Several options for applying anti-tumor cargo to MSC-derived exosomes include the loading with chemotherapeutics via electroporation or equipment with specific proteins, metabolites, or designed miRs for interference with tumor-regulatory pathways [49]. Other technical approaches revealed the ability of MSC to release extracellular vesicles after entrapping silk/curcumin nanoparticles using a so-called “carrier-in-carrier” system [50,51].

Advantages of MSC-derived exosomes include cell-specific tropism, an efficient biocompatibility and a reduced intrinsic immunogenicity among others, which characterizes them as an appropriate cell-free vehicle to deliver, e.g., vaccines, regenerative material, or anti-tumor cargo. Thus, MSC engineered for a suicide gene expression and delivery via exosomes demonstrated growth inhibition in human prostate or breast cancer cell lines in the presence of the prodrug 5-fluorocytosine [52]. Moreover, exosomes released by macrophages and loaded with paclitaxel by sonication were suggested as potential biological tool for delivery of a therapeutic compound [53]. Previous findings also demonstrated that paclitaxel-loaded exosomes from murine MSC or from human gingival papilla-derived MSC reduced in vitro growth of tumor cell lines [54,55]. Furthermore, MSC educated with photosensitizer-loaded nanoparticles and subsequent irradiation induced cell death in osteosarcoma cells during in vitro co-culture [56]. Other work introduced nanoparticle-engineered quantum dots in MSC as a promising drug delivery vehicle to tumor cells in a three-dimensional in vitro co-culture system [57]. An alternative and extended concept was followed in the present work by applying MSC-released taxol vesicles in an in vivo tumor system, demonstrating significant therapeutic effects both on the primary tumors and on distant organ metastases. Of interest, our intravenous application of exosome-associated drug delivery in vivo demonstrated a similar reduction in formation of metastases with a 1000-fold reduced taxol amount in exosomes as compared to the corresponding application of the substance itself, suggesting specific tumor cell addressing and accumulation paralleled by reduced degradation of taxol exosomes in the liver. Detection of approximately 10-fold higher amounts of taxol substance in the liver, as compared to kidney tissue, is supported by previous studies demonstrating predominantly hepatic metabolization of taxol via cytochrome P450 isozyme-mediated hydroxylation [58]. These findings suggest a highly specific tumor targeting by the MSC-derived exosomes. Indeed, selective tumor cell adhesion of paclitaxel-loaded exosomes was also demonstrated in pancreatic carcinoma cells by using an autologous in vitro system [59]. Our findings furthermore suggest a pronounced reduction of side effects when a markedly lower drug administration via specifically tumor-targeting exosomes achieves similar therapeutic effects in reduction of both tumor growth and the appearance of metastases.

## 4. Materials and Methods

### 4.1. Cell Culture

Isolation of primary human MSC was performed from umbilical cord explant cultures as reported previously [60]. The cells were cultured in αMEM (Sigma Chemie GmbH, Steinheim, Germany) supplemented with 10% of allogeneic human AB-serum (blood from 31 male AB donors was commercially obtained from a blood bank, Hannover Medical School, Germany, and processed to serum), 100 U/mL penicillin, 100 µg/mL streptomycin, and 2 mM L-glutamine (Sigma). Subculture of MSC was performed following accutase (Sigma) treatment for 3 min at 37 °C. MSC from four different donors and passages (MSC280416 P2; MSC290115 P3; MSC030816 P4; MSC060616 P6) were used in the experiments.

Human umbilical vein-derived endothelial cells (HuVECs) from two different donors were isolated and cultured in endothelial cell basal medium MV2 (PromoCell GmbH, Heidelberg, Germany) together with the endothelial cell growth medium supplement mix (PromoCell GmbH). Subculture of HuVECs was performed by treatment with Trypsin/EDTA solution (Sigma) for 10 min at 37 °C. 

The isolation of primary human cells from umbilical cord tissue has been approved by the local Ethics Committee of Hannover Medical School, Project #443 on February 26th, 2009. Informed written consent was obtained from all donors.

Human SK-OV-3 ovarian cancer cells (ATCC^®^ #HTB-77TM) were commercially obtained in P25 from the ATCC, Manassas, VA, USA, originally established from the malignant ascites of a patient with progressive adenocarcinoma of the ovary.

Human A549 lung carcinoma cells were used as reported elsewhere [61]. This cell line was originally derived from explanted alveolar basal epithelial adenocarcinoma of a 58-year-old Caucasian male. 

The ovarian and lung cancer populations were cultivated at 1750 cells/cm^2^ in RPMI 1640 supplemented with 10% (v/v) fetal calf serum, 2 mM L-glutamine, 100 U/mL penicillin, and 100 µg/mL streptomycin. Subculture was performed by trypsin/EDTA (Biochrom GmbH, Berlin, Germany) treatment for 5 min at 37 °C.

Human MDA-hyb1 breast cancer cells were cultured in MSC culture medium. This population represents an aggressively tumorigenic and highly metastatic breast cancer cell line derived from an MSC/MDA-MB-231 breast cancer cell fusion as characterized previously [25]. 

All cell lines were tested for mycoplasma by the luminometric MycoAlert Plus mycoplasma detection kit (Lonza Inc., Rockland, ME, USA) according to the manufacturer’s recommendations. Cell line authentication was performed by short tandem repeat (STR) fragment analysis using the GenomeLab human STR primer set (Beckman Coulter Inc., Fullerton, CA, USA) and was confirmed in previous work [62] according to the STR database provided by the ATCC, Manassas, VA, USA.

In order to detect the primary tumor and metastatic cells in vivo and to quantify proliferative capacity in the fluoroscan assay in vitro, the different MSC and tumor cell populations were transduced with a 3rd generation lentiviral SIN vector containing the eGFP or the mcherry gene, respectively, as described in previous work [14].

### 4.2. Flow Cytometry Analysis

For antibody staining, the cells were blocked with 2% FCS in PBS for 15 min at room temperature, washed with PBS, and stained with mouse monoclonal CD31-FITC (clone WM59, IgG1, Dako, Agilent, Santa Clara, CA, USA), mouse monoclonal CD90-PE (clone 5E10, IgG1, BioLegend, San Diego, CA, USA), and dual PE-/FITC-labeled IgG1 control antibody (Dako Denmark AS, Glostrup, Denmark) at 4 °C for 15 min, respectively. Following two washes with PBS, cells were analyzed in a Galaxy (Sysmex Partec GmbH, Münster, Germany) flow cytometer using FlowMax V2.9 software (Sysmex Partec GmbH).

### 4.3. Preparation of Control Exosomes and Chemotherapeutic-Loaded Exosomes

Subconfluent cell cultures (about 2 × 10^6^ cells) at a density of 1.4 × 10^4^ cells/cm^2^ were incubated either in appropriate dilution (1:700) of solvent (50.17% ethanol = final concentration of 0.07% ethanol) for control exosomes or in the presence of 10 µM taxol (paclitaxel in solvent, diluted 1:700 from a 7 mM stock solution) for 24 h, washed with serumfree culture medium, and incubated with serumfree culture medium for a further 24 h. Thereafter, the conditioned medium was removed and the supernatant was sequentially centrifuged in four steps (1. 360 *g* for 10 min to remove cells; 2. 2000 *g* for 10 min to remove dead cells; 3. 10,000 *g* for 30 min to remove debris and large vesicles; 4. 100,0000 *g* for 70 min to precipitate exosome-like particles) according to the protocol by Thery et al. [63]. While some characterization for exosomal properties was performed according to the recently updated MISEV (minimal information for studies of extracellular vesicles) 2018 standards [64], the obtained vesicles were termed exosomes in this manuscript rather than EVs although no further purification was performed after the four centrifugation steps. Aliquots of cellular protein and of the corresponding exosomes were quantified by measurement of protein concentration using the colorimetric BCA-assay (Thermo Scientific, Schwerte, Germany). The precipitated cell-derived exosomes were resuspended in 50 µL of PBS and stored at −80 °C. 

### 4.4. Characterization of Exosome Preparations by Nanoparticle Tracking Analysis (NTA)

The different exosome preparations in PBS were analyzed for vesicle concentration, size distribution, and preparation quality in scatter and fluorescence mode using the ZetaView PMX120 NTA (Particle Metrix GmbH, Meerbusch, Germany) with an embedded 40 mW laser at 488 nm and a CMOS camera. Zeta potential was measured using 0.05 × PBS to adjust conductivity to approximately 500 uS/cm. For each measurement, one cycle was performed by scanning 11 randomly chosen positions of particle distribution and by capturing 60 frames in scatter mode per position and 15 frames in fluorescence mode per position using the following settings: camera sensitivity in scatter mode: 82.0; camera sensitivity in fluorescence mode: 97.0; shutter: 100. Following frame capture, the videos were analyzed by the in-build ZetaView Software 8.05.05. SP2 (Particle Metrix GmbH) with appropriate analysis parameters: maximum particle size: 1000, minimum particle size: 5, minimum particle brightness: 30; tracelength: 15 in scatter mode and 7 in fluorescence mode.

### 4.5. Immunoblot Analysis of Exosomes

Western blot analysis was performed as described previously [65]. Briefly, protein amounts of exosomal preparations were measured using the BCA method and 10 µg of exosomal proteins were separated on a 10% SDS polyacrylamide gel and transferred to a nitrocellulose membrane (GE Healthcare Lifescience, Freiburg, Germany) after semi-dry blotting (Peqlab Biotechnology GmbH, Erlangen, Germany) at 1.5 mA/cm^2^ for 1 h. The blots were incubated with a 1:200 dilution, respectively, of the mouse monoclonal antibody CD63 (clone MX-49.129.5) (Santa Cruz Biotechnology, Inc., Heidelberg, Germany) 

### 4.6. Detection and Quantification of Taxol in Culture Medium, Cells, and Exosomes by Liquid Chromatography-Tandem Mass Spectrometry (LS-MS/MS) Analysis

Corresponding aliquots of the 24 h taxol cell culture medium supernatants were treated with ice cold extraction solvent and simultaneously, the four MSC cultures treated with 10 µM taxol for 24 h were washed with PBS and lysed with 700 µL of ice cold extraction solvent (acetonitrile–methanol–water (2:2:1, v/v); J.T. Baker., Deventer, The Netherlands), respectively. Likewise, exosomes isolated from the four taxol-treated MSC populations were extracted with ice cold extraction solvent, respectively. Aliquots of tumor tissues and organ metastases were extracted with 800 µL of ice cold extraction solvent with 0.25 μM docetaxel (Sigma and Aldrich, St. Louis, MO, USA) as an internal standard.

All extracts were frozen overnight at −20 °C to complete protein precipitation. Thereafter, the samples were centrifuged for 10 min at 20,800 *g* at 4 °C. The supernatant fluid was transferred to a 2 mL reaction tube and evaporated under a gentle nitrogen stream at 40 °C. The dried pellet was reconstituted in 100 µL of internal standard solution. Samples were again centrifuged for 10 min at 20,800 *g* at 4 °C, and the supernatant fluid was transferred to a mass spectrometry vial. Aliquots of 10 µL were injected into the LC-MS/MS system.

As calibrators, 100 µM stock solutions of paclitaxel were prepared in methanol (J.T. Baker) and 10 calibrators were prepared by an appropriate 1:2 dilution, resulting in a calibration range from 0.26 to 1000 nM paclitaxel. Calibrators were stored in 50 µL aliquots at −20°C. An internal standard solution was prepared in HPLC-grade water (J.T. Baker) with docetaxel (Sigma) to a final concentration of 1 µM. Each calibrator was treated with 200 µL of ice-cold extraction solvent, and the mixture was dried under a constant nitrogen stream at 40 °C. The dried pellet was dissolved in 100 µL of internal standard solution.

Culture medium-, tissue-, cell-, and exosome-extracted paclitaxel and calibrators were analyzed by LC-MS/MS using a Shimadzu HPLC-system (Shimadzu, Duisburg, Germany), consisting of two HPLC-Pumps (LC-30AD), a temperature controlled autosampler (SIL-30AC), a degasser (DGU-20A4), and a column oven (CTO-20AC). For chromatographic analysis, a C18 reversed phase column (Zorbax Eclipse XDB-C18, 4.6 × 50 mm (Agilent Technologies, Santa Clara, CA, USA) was used. This column was connected to a C18-Security guard (Phenomenex, Aschaffenburg, Germany) and a 2 µ column saver (Sigma-Aldrich). The column was maintained at 25 °C. Mobile phases were water (A) and methanol (B), each containing 0.1% formic acid. The initial solvent composition was set to 50% A and 50% B. For analyte separation, a gradient was applied starting with an increase of solvent B to 95% within 6.9 min. This solvent composition was kept for 5 min before the column was re-equilibrated to starting conditions for 4 min. Total analysis runtime was 16 min with a flow rate of 0.4 mL/min. Under these conditions, retention times of 6.3 min (paclitaxel) and 6.5 min (docetaxel, internal standard) were observed.

Detection and quantification of paclitaxel was carried out on a tandem mass spectrometer (5500QTRAP^®^ (Sciex, Framingham, Massachusetts) equipped with an ESI-source (electrospray ionization). For positive SRM detection, mass transitions for paclitaxel and docetaxel were identified as follows: paclitaxel: m/z 854→105 (quantifier) and m/z 854→286 (identifier); docetaxel (internal standard): m/z 808→226. Control of LC and the mass spectrometer as well as data sampling was performed by Analyst software (version 1.5.2., Sciex). For quantification, calibration curves were created by plotting peak area ratios of paclitaxel, and the internal standard versus the nominal concentration of the 10 calibrators. The calibration curve was calculated using quadratic regression and 1/× weighing. The lower limit of quantification (LLOQ) was defined as the lowest calibrator point, showing an accuracy of 100 ± 20% regarding the nominal concentration. For paclitaxel, an LLOQ of 0.24 nM was calculated.

### 4.7. Cytotoxicity Measurements of Exosomes by Fluoroscan Assay

Control and taxol-loaded exosomes from MSC and HuVECs were dissolved in appropriate cell growth medium and different dilutions were incubated with in vitro cultures of different cancer cell populations. The proliferative capacity was evaluated by fluorescence measurement using the fluoroscan assay as previously described [61]. Briefly, 1000 A549^GFP^, SK-OV-3^GFP^, or MDA-hyb1^cherry^ cells/well were seeded with standard culture medium (100 μL/well) in flat bottom 96-well plates (Nunc/ThermoFischer Scientific, Roskilde, Denmark) and incubated overnight to allow attachment. Thereafter, 100 µL of culture medium with drug solvent was added to the cells as a control, and in other wells 100 µL of culture medium containing appropriate dilutions of taxol substance, taxol-loaded exosomes, and control exosomes were respectively added to the cells. Following incubation for 72 h, the medium was removed and the cells were lysed with 5% (w/v) SDS. Afterwards, the fluorescence intensities of GFP or cherry in the cell homogenate, which corresponded to the appropriate cell number of cancer cells, were measured at an excitation of 485 nm and an emission of 520 nm (GFP), or an excitation of 584 nm and an emission of 612 nm (cherry) using the Fluoroscan Ascent Fl (ThermoFisher Scientific, Schwerte, Germany). 

### 4.8. Cell Cycle Analysis

Detection and quantification of the different cell cycle phases in the various MSC cultures was performed as described previously [66]. Briefly, 10^5^ cells were fixed in 70% (v/v) ice-cold ethanol at 4 °C for 24 h. Thereafter, the fixed cells were stained with propidium iodide staining solution (12.5 µg/mL propidium iodide, 0.5% Triton-X-100 and 100 U/mL DNase-free RNase in PBS). The samples were then analyzed in a FACSCalibur (BD Biosciences GmbH, Heidelberg, Germany) flow cytometer using the FlowJo V10 cell cycle software.

### 4.9. In Vivo Experiments

Animal research using NODscid mice was carried out by following the internationally recognized guidelines on animal welfare and has been approved by the institutional licensing committee ref. # 33.19-42502-04-15/1992 on Dec. 18th, 2015.

About 2 × 10^6^ MDA-hyb1 cells were injected subcutaneously into 12 animals of 5–6 weeks old female NODscid mice, respectively. After 5 days post-injection, all 12 mice had developed subcutaneous tumors. The tumor-bearing mice were randomized into three groups, and treatment was started by intravenous application of a 100 µL volume of MSC-derived control exosomes, taxol-loaded exosomes, or taxol substance, as follows: 

1. intravenous application of MSC-derived control exosomes twice weekly (→ control tumor development);

2. intravenous application of subsequently derived exosomes from taxol-treated MSC twice weekly;

3. intravenous application of initially 10 mg/kg taxol and thereafter, due to weight loss and incompatibility, 5 mg/kg taxol twice weekly.

Tumor progression was monitored and measurement of tumor size was recorded using a digital caliper (VWR International). 

After 21 days post-MDA-hyb1 cell transplantation when control tumors of the four animals treated with MSC-derived control exosomes exceeded 2 cm^3^ and thus, reached criteria for termination of the experiment, all animals were sacrificed by cervical dislocation. Primary tumor tissues were dissected, washed in PBS, and weighted. Organs were also dissected from the mice, and thin sections were analyzed by fluorescence microscopy for the presence and accumulation of metastatic cells.

### 4.10. Transcript Analysis by RT-PCR

Total RNA was isolated from the tumor tissues and the organs using RNeasy Mini Kit (Qiagen, Hilden, Germany) according to the manufacturer’s instructions. One µg of RNA was reverse-transcribed into cDNA, and reactions were performed with corresponding primers specifically as described previously [25].

## 5. Conclusions

In summary, MSC-derived taxol exosomes exhibited superior cytotoxic in vitro effects when compared to taxol exosomes from HuVECs. Moreover, these data demonstrate promising therapeutic in vivo effects of taxol-treated MSC-derived exosomes, which was associated with a marked inhibition of primary tumor growth and a reduction of distant organ metastases to the same level observed with an about 1000-fold higher treatment using taxol substance. While systemic exosome application was very well tolerated by the mice, this approach provides encouraging future perspectives including a possible combination of different chemotherapeutics via MSC-educated exosomes or the use of further exosome sources for more specific addressing of tumor cells. Moreover, drug-loaded MSC-derived exosomes may also provide opportunities to target heterogeneity of the tumor including the presence of cancer stem-like cells.

## Supplementary Materials

The following are available online at https://www.mdpi.com/2072-6694/11/6/798/s1, Figure S1: MSC-derived exosomal preparation is documented by transmission electron micrographs demonstrating a single rounded exosome of approx. 90 nm in diameter (lower left panel). The content of MSC-secreted exosomes includes among others proteinous precipitate (indicated by arrows, upper left and right panel). Exosomes isolated from MSC cultures are varying in size between 50 to 200 nm (right panel). Bar represents 200 nm. Figure S2: Characterization of HuVECs by FACS analysis using the PE-labeled CD90 and the FITC-labeled CD31 antibodies. Figure S3: The average diameter of exosomes isolated from 2 different HuVEC control cultures (light blue bars) and corresponding 2 HuVEC cultures after treatment with 10 µM taxol for 24 h (light red bars) was measured by NTA using the ZetaView PMX120. Figure S4: Exosome concentration of two different HuVEC control cultures (light blue bars) and corresponding two HuVEC cultures after treatment with 10 µM taxol for 24 h (light red bars) was evaluated by NTA measurement. Figure S5: The original Western blot is demonstrated according to the data in Figure 2C, upper panel.
