# Supplementary material for: Taxol-Loaded MSC-Derived Exosomes Provide a Therapeutic Vehicle to Target Metastatic Breast Cancer and Other Carcinoma Cells
Source: Cancers (Basel). 2019 Jun 9;11(6):798. doi: 10.3390/cancers11060798 (PMC6627807; doi:10.3390/cancers11060798)
Supplement: Supplementary file 1 [file cancers-11-00798-s001.pdf]

Supplementary Materials: Taxol-Loaded MSC-Derived Exosomes Provide a Therapeutic Vehicle to Target Metastatic Breast Cancer and Other Carcinoma Cells

Catharina Melzer, Vanessa Rehn, Yuanyuan Yang, Heike Bahre, Juliane von der Ohe, and Ralf Hass

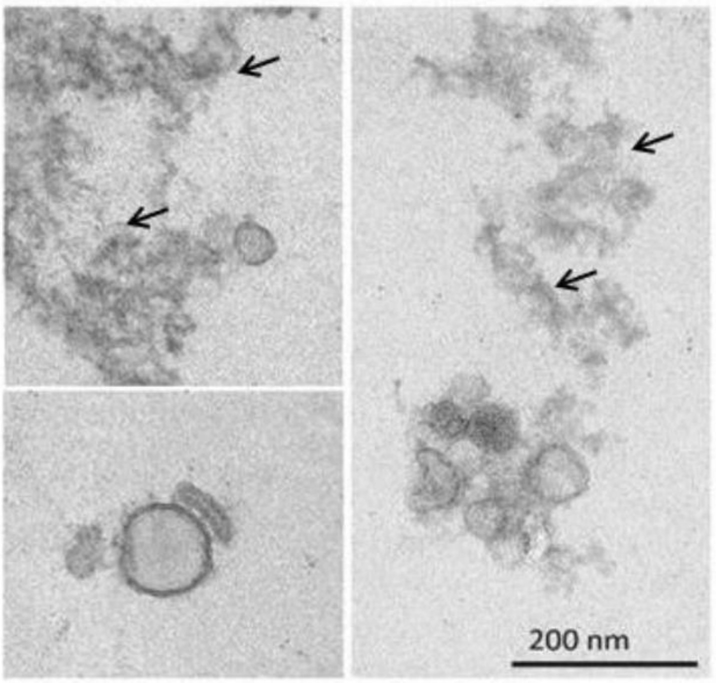

Figure S1. Transmission electron micrographs of MSC-derived exosome preparation by ultracentrifugation method and subsequent 1.5% formaldehyde/1.5% glutaraldehyde fixation was performed as described elsewhere [46] and demonstrate a single rounded MSC-derived exosome of approx. 90 nm in diameter (lower left panel). The content of MSC-secreted exosomes includes among others proteinous precipitate (indicated by arrows, upper left and right panel). Exosomes isolated from MSC cultures are varying in size between 50 to 200 nm (right panel). Bar represents 200 nm.

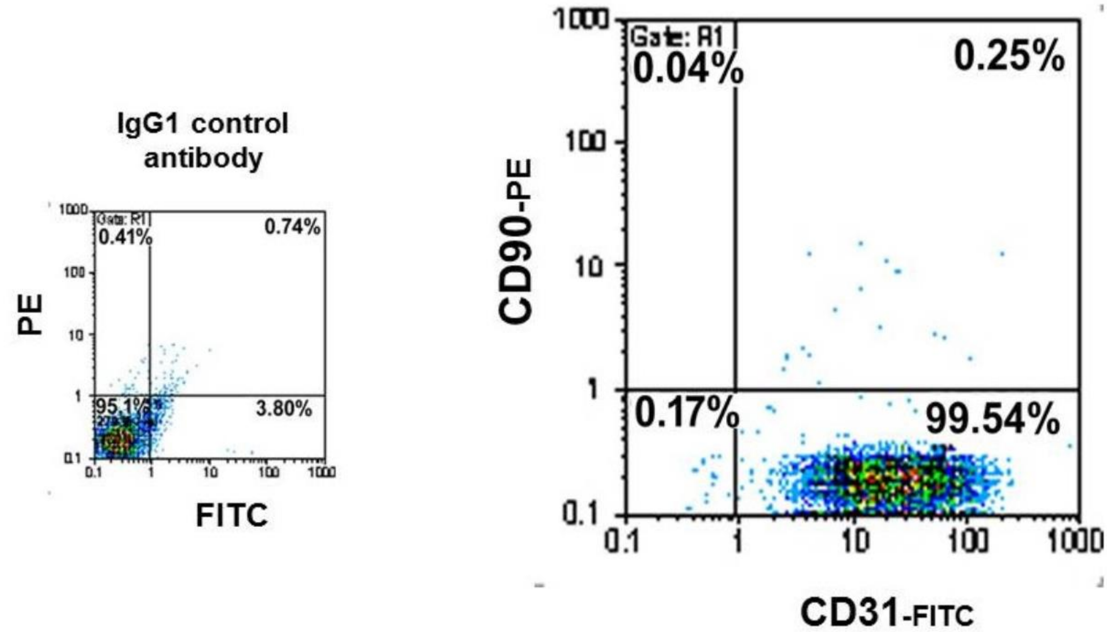

Figure S2. Characterization of HuVECs by FACS analysis using the PE-labeled CD90 and the FITC-labeled CD31 antibodies. A dual PE-/FITC-labeled IgG1 antibody served as control.

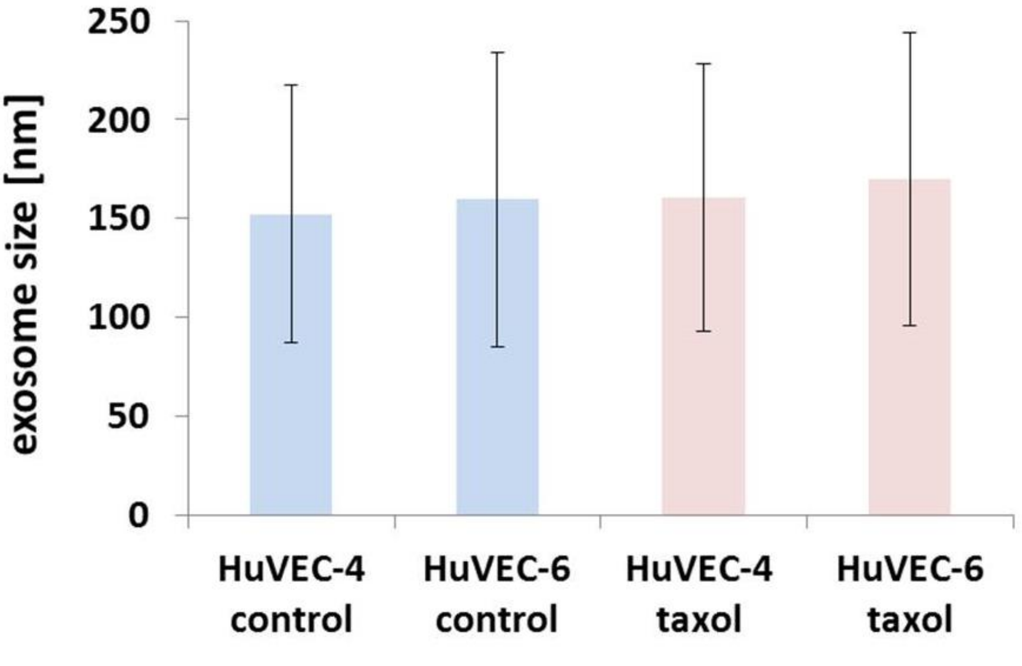

Figure S3. The average diameter of exosomes isolated from 2 different HuVEC control cultures (light blue bars) and corresponding 2 HuVEC cultures after treatment with 10 µM taxol for 24 h (light red bars) was performed by NTA measurement using the ZetaView PMX120.

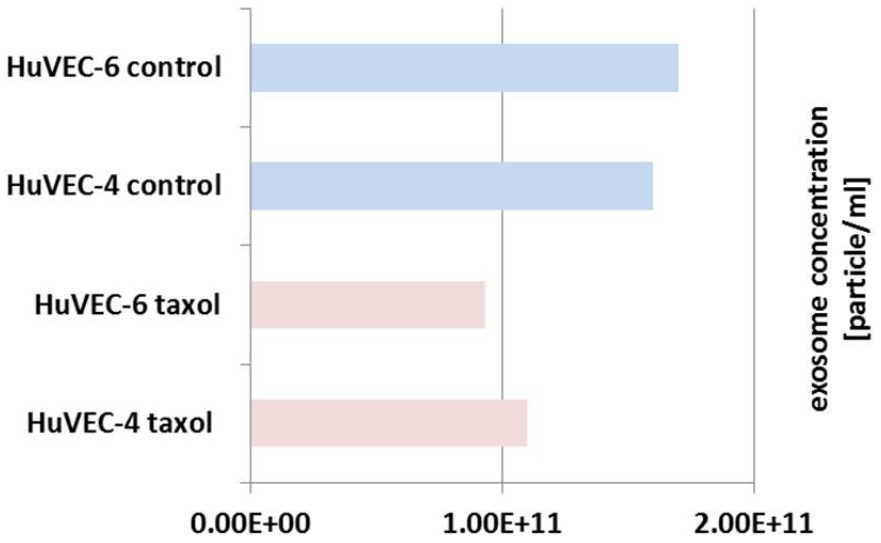

Figure S4. The exosome concentration of 2 different HuVEC control cultures (light blue bars) and corresponding 2 HuVEC cultures after treatment with 10 μM taxol for 24 h (light red bars) was evaluated by NTA measurement.

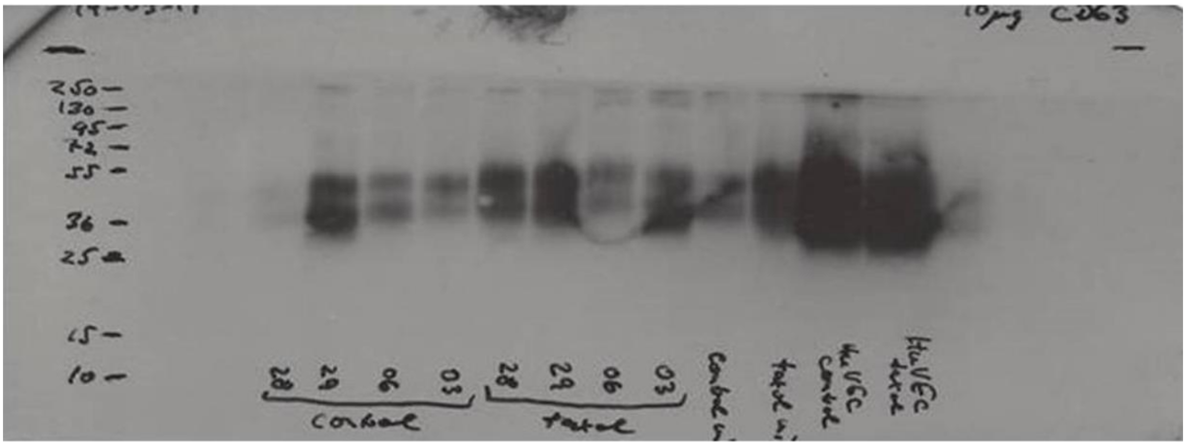

Figure S5. Original Western blot is demonstrated according to the data in Figure 2C, upper panel.
